# Friend Network Growth Through Social Activities: A Longitudinal Analysis of Participation in Specific Activities

**DOI:** 10.1093/geroni/igaf122.2991

**Published:** 2025-12-31

**Authors:** Haosen Sun

**Affiliations:** University of Nevada, Reno, Reno, Nevada, United States

## Abstract

Engaging in social activities and maintaining broader, more diverse social networks help to mitigate loneliness and support cognitive health in older adults. However, which activities (volunteering, courses, clubs, organizations, and interactive games) are more effective in fostering new friend connections for intimate discussions—while accounting for changes in participation over time—remains unclear. We use Wave 4 (2011) and Wave 6 (2015) of the Survey of Health, Aging and Retirement in Europe (SHARE), to conduct negative binomial regression with a longitudinal change score framework. Continued monthly participation in social activities is consistently associated with adding more new friends, with clubs and organizations showing the strongest effects. Starting to participate also shows a significant positive effect, particularly for clubs and interactive games where the magnitude exceeds that of continued participation. However, for other activities, starting participation tends to have similar or weaker effects than sustained engagement. Surprisingly, quitting activities is also often positively associated with having more new friends, although the magnitude is generally smaller. Meanwhile, quitting courses and games does not show a significant positive association. Sustained participation in social activities, particularly in clubs and organizations, significantly contributes to bringing more friends into core discussant network. Starting new activities—especially shared-interest-based ones like clubs and games—is also effective. While quitting activities is often accompanied by active adjustments in one’s social convoy, more attention is needed for individuals leaving courses or games, as these activities tend to be more structured and based on fixed membership.

